# REGNET: mining context-specific human transcription networks using composite genomic information

**DOI:** 10.1186/1471-2164-15-450

**Published:** 2014-06-09

**Authors:** Sang-Mun Chi, Young-Kyo Seo, Young-Kyu Park, Sora Yoon, Chan Young Park, Yong Sung Kim, Seon-Young Kim, Dougu Nam

**Affiliations:** School of Computer Science and Engineering, Kyungsung University, Busan, Republic of Korea; School of Life Sciences, UNIST, Ulsan, Republic of Korea; Medical Genomics Research Center, Korea Research Institute of Bioscience and Biotechnology, Daejeon, Republic of Korea; Division of Mathematical Sciences, UNIST, Ulsan, Republic of Korea

**Keywords:** Composite gene-set analysis, Microarray, Transcription network, TFBS, Gene Ontology, KEGG

## Abstract

**Background:**

Genome-wide expression profiles reflect the transcriptional networks specific to the given cell context. However, most statistical models try to estimate the average connectivity of the networks from a collection of gene expression data, and are unable to characterize the context-specific transcriptional regulations. We propose an approach for mining context-specific transcription networks from a large collection of gene expression fold-change profiles and composite gene-set information.

**Results:**

Using a composite gene-set analysis method, we combine the information of transcription factor binding sites, Gene Ontology or pathway gene sets and gene expression fold-change profiles for a variety of cell conditions. We then collected all the significant patterns and constructed a database of context-specific transcription networks for human (*REGNET*). As a result, context-specific roles of transcription factors as well as their functional targets are readily explored. To validate the approach, nine predicted targets of E2F1 in HeLa cells were tested using chromatin immunoprecipitation assay. Among them, five (*Gadd45b*, *Dusp6*, *Mll5*, *Bmp2* and *E2f3*) were successfully bound by E2F1. c-JUN and the EMT transcription networks were also validated from literature.

**Conclusions:**

REGNET is a useful tool for exploring the ternary relationships among the transcription factors, their functional targets and the corresponding cell conditions. It is able to provide useful clues for novel cell-specific transcriptional regulations. The REGNET database is available at http://mgrc.kribb.re.kr/regnet.

**Electronic supplementary material:**

The online version of this article (doi: 10.1186/1471-2164-15-450) contains supplementary material, which is available to authorized users.

## Background

Cells alter the process of transcriptional regulation so as to adjust to or drive changes in the cellular conditions between different stages of the cell cycle, stem cell differentiation or cancer development. Such changes in the transcriptional networks between different cell conditions are represented in the transcriptome data that are obtained using microarrays or high-throughput sequencing. However, elucidating the complex transcription networks (TNs) has been a daunting task in spite of the remarkable advances in both computational modeling and high-throughput experimental technologies [1–8]. One main reason for the difficulty is the dynamic nature of the networks: Transcription factors (TFs) not only regulate different targets depending on the cell conditions, but their effects on these targets can also change. Therefore, statistical models that estimate the average connectivity of the networks from a large collection of transcriptome data may confer limited accuracy.

Taking these issues into account, we have developed an approach for identifying the context-specific TNs from a large collection of gene expression fold-change profiles. Toward this end, we collected 2,482 paired (test/control) human microarray datasets encompassing a variety of cell conditions. Using a composite gene-set analysis method (ADGO) [9, 10], we combined the information of TF binding site (TFBS), Gene Ontology or KEGG pathway gene sets, and the gene expression fold-change profiles, and thereby tried to address the fundamental but largely open question of which TFs regulate which functions or pathways under differing cell conditions?

**Figure 1:**
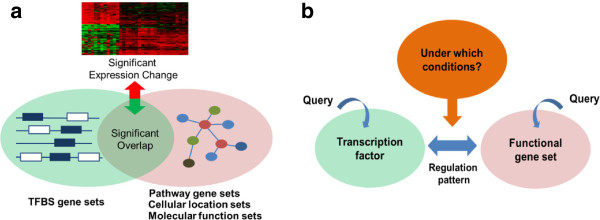
**Overview of REGNET. (a)** Association of a TF and functional gene set. **(b)** Ternary relationships explored by REGNET.

Since the majority of TFBSs are potentially false positives, we applied the following filtering criteria to identify reliable TNs as follows. (A) A *TFBS gene set* (genes that share a common TFBS in their promoters) is required to have a significant overlap with a *functional gene set* (genes that share a common annotation in GO or KEGG). (B) The genes that overlap, as a whole, should exhibit significant expression changes. (C) Such changes should be observed across many microarray conditions (e.g. ten or more). If the above three criteria are satisfied between a TFBS gene set and a functional gene set, we say the corresponding TF is *associated* with the functional gene set (Figure 1a), and the associated pairs as well as the corresponding conditions are output as context-specific TNs.

We collected all of the 12,149,291 significant triples (TF, functional gene set, condition; q-values for the two conditions (A) and (B) <0.05) in order to construct a context-specific TN database, dubbed *REGNET*. It is used for exploring the ternary relationships among the TFs, their functional targets and the corresponding cell conditions (Figure 1b). Each TF or function (or pathway) name can be used as a query, and REGNET provides corresponding context-specific TNs. Using this database, we analyzed TNs for two TFs, E2F1 and c-JUN. Many known target genes as well as corresponding microarray conditions were identified. REGNET also included many predictions for novel targets. For example, we selected nine candidate targets of E2F1 predicted for HeLa cells and tested them using a ChIP assay. Among them, five (*Gadd45b*, *Dusp6*, *Mll5*, *Bmp2* and *E2f3*) were successfully bound by E2F1. We also validated the TNs for c-JUN and the ‘epithelial to mesenchymal transition (EMT)’ gene set from the literature. REGNET is available at http://mgrc.kribb.re.kr/regnet.

## Methods

### Collection of data

Human gene expression microarray datasets of the same platform ‘HG-U133 Plus 2’ were downloaded from 839 Gene Expression Omnibus [11] series. In each series dataset, we manually identified the test/control sample groups to collect log fold-change profiles. Many datasets contained multiple test groups, and hence 2,482 fold-change profiles were collected in total. Probe values that correspond to the same gene were averaged to yield gene-based fold-change profiles (20,361 genes). human TFBS gene sets were obtained from MSigDB [12]. For functional gene sets, the three categories of GO and KEGG pathways were used. GO gene sets were obtained from a gene product association file downloaded from a ftp site (http://www.geneontology.org/gene-associations/). Both the electronic and non-electronic annotations were used to maximize the coverage. KEGG gene sets were downloaded from MSigDB. All the offspring terms are again included in their parent terms. All the gene-sets with not less than 10 and not more than 500 genes are included in the analysis and the system.

### Identification of context-specific transcription networks

We applied the following three filtering criteria to identify reliable TNs:

(A) Overlap significance: the significance of overlap for every pair of a TFBS gene set and functional gene set is assessed using the hypergeometric distribution. The genes that overlap, if significant (default: FDR q-value < =0.02), are regarded as candidate targets of the TF, and may be interpreted as the channel through which the TF regulates the function (or pathway). Such overlapping sets constitute the candidate TNs to be further examined throughout gene expression datasets.

(B) Expression significance: For each pair of TFBS-functional gene sets that overlap significantly, another filtering criterion using a fold-change expression dataset is applied: The Z-statistic on the gene expression fold-change values is computed for each of the TFBS set, functional set and their overlapping set, respectively. If the FDR q-value of the overlapping set is less than or equal to 0.02 (default value) and the q-values for the two individual sets are larger than 0.05 (default value), we assume the corresponding TN is activated on the given microarray condition. In other words, expression changes in the overlapping genes should be pronounced compared to the two individual sets [9].

(C) Number of conditions: Only TNs that satisfy the above two criteria (A) and (B) across *k* (default number =10) or more microarray conditions are regarded as reliable transcription patterns.

**Figure 2:**
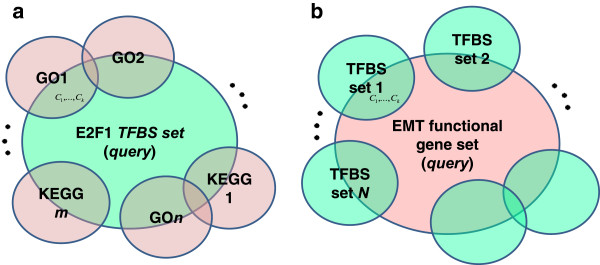
**Context-specific transcription networks for the query of a TF or functional gene set. (a)** For the query of a TF, multiple functional gene sets are associated. *C*_*l,*_ *C*_2, …,_ *C*_*k*_ represent the microarray conditions of the fold-change profiles for which the genes in the overlap parts significantly altered their expressions. The collection of the genes in the overlap parts are used for a global assessment. **(b)** For the query of a functional gene set, multiple TF’s are associated.

If all these criteria are satisfied between a TFBS gene set and functional gene set, the TF, candidate targets as well as the corresponding conditions are output as context-specific TN. Among the overlap set identified through (A) ~ (C), we can further select individual candidate targets that show some high fold expression changes (e.g. ±1.5 of higher) across a number of conditions (e.g., more than 0.3 ×*k* conditions). All the threshold values including q-values and *k* can be changed by the user for more thorough exploration of the transcriptional regulations. Figure 2 summarizes the context-specific TNs queried by TF and functional gene set, respectively.

### Chromatin Immunoprecipitation (ChIP) assay for E2F1

ChIP assays for E2F1 in HeLa cells were performed following the protocol represented at http://genomics.ucdavis.edu/farnham. HeLa cells were crosslinked for 10 min by adding formaldehyde to a final concentration of 1% with mild agitation. Crosslinking was stopped by the addition of glycine to a final concentration of 125 mM, and cells were washed three times with ice-cold PBS prior to harvesting by scraping of the plates. Chromatid were fragmented for 20 min (10 sec on pulse and 20 sec off pulse) to produce fragments ~500 nt in size using the Bioruptor sonicator (Diagenode). Antibody (SC251X; Santa Cruz Biotech) to E2F1 was used to pull down target chromatid from 1 × 10^8^ cells. Genomic DNAs were isolated from proteinase K-treated (45°C for 1 h) samples and purified. ChIP samples were tested by PCR using positive and negative control primer sets shown in Additional file 1: Table S1. The quantitative real-time PCR for ChIP is described in Supplementary Material.

## Results

### REGNET database

**Figure 3:**
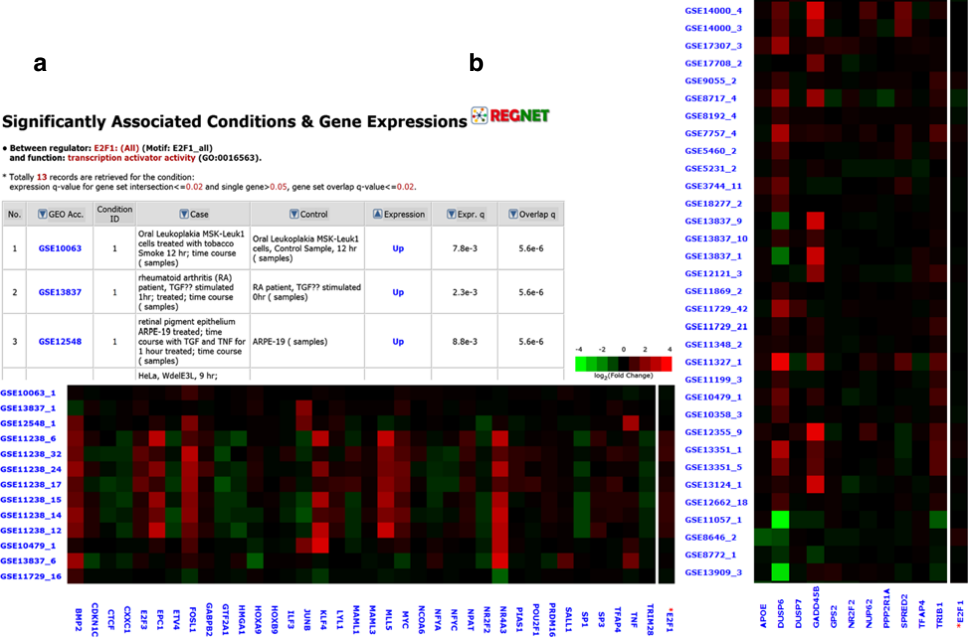
**Condition table and heat maps for transcription networks of E2F1. (a)** The condition table and heat map for the association between E2F1 and the gene set ‘transcription activator activity (GO:0016563)’. Several targets exhibit clear activations across a number of virus treated HeLa cells. Seven genes, *Bmp2*, *E2f3*, *Epc1*, *Fosl1*, *Mll5*, *Myc* and *Nr4a3* are chosen for validation using a ChIP assay. **(b)** The heat map for the association between E2F1 and the gene set ‘negative regulation of protein kinase activity (GO:0006469)’. A part of the 55 conditions that associate E2F1 and the GO gene set are shown. Among the targets, *Gadd45b* and *Dusp6* exhibit clear expression changes across a variety of microarray conditions. Both genes are tested using a ChIP assay.

REGNET is an intuitive and easy to use database developed for exploring context-specific TNs (http://mgrc.kribb.re.kr/regnet). REGNET accepts two kinds of queries, i.e. TF and function (or pathway, localization) names, respectively. It provides a webpage presenting all the TF names analyzed, so that investigators can simply choose the TF of their interest. Each TF name indicates a TFBS gene set to which the TF can bind. Because a TF can have different TFBSs, many of the TF names are represented multiple times. Among them, ‘(TF name)_all’ is linked to the TNs that can be captured only by taking the union of all the TFBS sets of their common TF. For example, AHR_all links to the TNs that can be captured only by using the union of the four different TFBS sets (V$AHRARNT_01, V$AHRARNT_02, V$AHR_01 and V$AHR_Q5).Each TF name is linked to a list of functional gene sets associated with the TF. Each line on the list is then linked to the corresponding target genes, descriptions for the TFBS and functional gene sets and the microarray conditions under which the target genes exhibit significant changes in expression. The ‘number of conditions’ leads to a table of the corresponding test/control microarray conditions. At the bottom of the table, a heat map of the log fold-change values of the target genes is displayed, summarizing the information for the associated pair of the TF and functional gene set (Figure 3). From this heat map, investigators can easily identify candidate targets that exhibit large expression changes across a number of conditions. The fold-change values of the TF itself are also provided on the right-end column of the heat map, for the activity of certain TFs can be usefully inferred from their transcript levels.

When querying for a functional gene set, a keyword is required for searching. For example, if the investigators want to identify TNs related to the ‘epithelial to mesenchymal transition’ process, entering ‘mesen’ is sufficient. Then, the names of functional gene sets that contain ‘mesen’ are all displayed. From among them, investigators can choose the exact gene set of their interest, and then all the TFs associated with that functional gene set are listed. As in the case of the TF query, each line is linked to detailed information on the target genes, TFBS sets and the queried gene set as well as the corresponding microarray conditions. More detailed information on the REGNET database may be seen from our web site.

### Analysis results for E2F1

We used the human TFBS gene sets from the Molecular Signatures Database (MSigDB) [12]. The MSigDB provides five E2F1 TFBS sets. The predicted results for E2F1 are accessible by choosing ‘E’ in the ‘TF-based browsing’ webpage. As expected, many cell-cycle related functional gene sets were found to be related to most of the TFBS gene sets. For example, the ‘DNA replication (GO:0006260)’ and ‘G1/S transition of mitotic cell cycle (GO:0000082)’ are associated with four of the five TFBS sets while the ‘cell cycle (KEGG)’ and ‘cell cycle checkpoint (GO:0000075)’ are associated with three. For each gene set, many conditions likely to activate cell cycle progression were observed, e.g. iPS or diverse cancer cell conditions.

For the union of all the E2F1 TFBS gene sets (E2F1_all), the ‘transcription activator activity (GO:0016563)’ set is captured and several of the putative targets exhibit high expression fold-changes for a number of the HeLa cell conditions treated with vaccinia virus (GSE 11238): *Bmp2*, *E2f3*, *Epc1*, *Fosl1*, *Mll5*, *Myc* and *Nr4a3* (Figure 3a). Another target set, ‘negative regulation of protein kinase activity (GO:0006469)’ is found to be associated with the E2F1_all set through 55 conditions. Among the targets, in particular, *Dusp6* and *Gadd45b* exhibit high expression fold-changes across dozens of heterogeneous conditions (Figure 3b). Although these 55 conditions do not include the HeLa cells, it was found that both genes display more than two-fold increases in their expression levels in most of the virus-treated HeLa conditions (GSE 11238).

**Figure 4:**
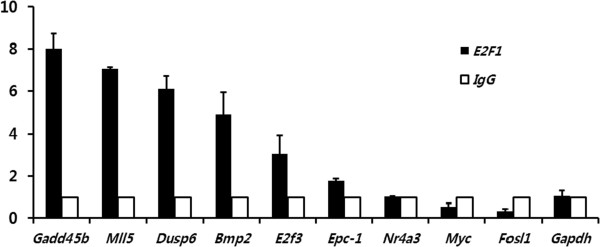
**Chromatin IP experimental results for E2F1.** The nine candidate targets of E2F1 were chosen for validation by gene-specific ChIP. The fold change (FC) is the fold increase of the signal from E2F1 antibody (black)–enriched chromatin relative to a control IgG (open). The negative control *Gapdh* exhibited no enrichment.

To validate the regulatory relationships, therefore, we performed a ChIP assay on HeLa cells, as described in Methods, for the nine candidate targets selected from the two associated sets ‘transcription activator activity’ and ‘negative regulation of protein kinase activity’. To this end, we activated E2F1 via the CDK4-Rb-E2F1 pathway [13] as described in Additional file 1: Figure S1. We used *Gapdh* as a control. The ChIP results are shown in Figure 4. Among the targets tested, five exhibited positive results: The binding of E2F1 to *Gadd45b*, *Dusp6* and *Mll5* was pronounced with six or higher fold-changes compared to a control IgG, and the binding to *Bmp2* and *E2f3* exhibited approximate five and three-fold increases, respectively.

### Analysis results for c-JUN

The MSigDB provides eleven c-JUN TFBS sets. The predicted results for c-JUN are accessible by choosing ‘J’ in the TF-based browsing webpage. The first TFBS set named ‘TGANTCA_V$AP1_C’ contains 866 genes, and 129 GO or KEGG gene sets are associated with this TFBS set as the result of satisfying the three filtering criteria (A) ~ (C). Among them, we identified two cell conditions for which c-JUN targets are most clearly activated. For example, the ‘response to steroid hormone stimulus (GO:0048545)’ gene set is associated with the c-JUN TFBS set for 56 microarray conditions. Among them, the HeLa cells treated with vaccinia virus (GSE 11238) clearly exhibits activation in five targets: *Adm*, *F3*, *Fosl1*, *Il6* and *Thbs1* (Additional file 1: Figure S2a). The latter four genes are known targets of c-JUN [14, 15]. *Thbs1* is known to be repressed by c-JUN in rat embryo fibroblasts [16], but it is also known to be activated by c-JUN in human hepatocarcinoma cell lines [15]. ADM is reported to induce the phosphorylation of c-JUN in glioblastoma cells [17], but is also a candidate target of c-JUN in our prediction. Because the known targets of c-JUN as well as *Jun* itself display high expression fold changes, we infer that viral infection to HeLa cells strongly increases the activity of c-JUN. In the ‘pathways in cancer (KEGG)’ gene set, we found another group of known c-JUN targets, i.e. *Il6*, *Lamb3*, *Lamc2*, *Mmp1* and *Mmp9*[14], that are strongly activated for the conditions of ‘engineered human skin’ (GSE 17539) [18] and ‘low dose treatment of 5-aza-2-deoxycytidine on an non-small cell lung cancer cell line’ (GSE 6695) [19] (Additional file 1: Figure S2b). If another TFBS set V$AP1FJ_Q2 is chosen, the well-known c-JUN interacting partner *Fos* as well as Il6 are captured in the ‘pathways in cancer (KEGG)’ gene set, and *Fos* exhibits a very strong co-expression pattern with *Jun* in many of the virus-treated HeLa cell conditions (Additional file 1: Figure S2c).

Summarizing the results for all the c-JUN TFBS gene sets, in total 156 functional gene sets are associated with at least one of the eleven TFBS gene sets. Of them, 52 functional gene sets (approximately 33%) are associated with two or more. This suggests that c-JUN binds to a variety of different TFBSs in order to ensure the regulation of its target pathways. For example, ‘pathways in cancer (KEGG)’ is associated with eight different c-JUN TFBS gene sets, highlighting the role of c-JUN as a key factor of cancer progression. The representative gene sets associated with three or more c-JUN TFBS sets are shown in Table 1.

**Table 1:** **Functional gene sets associated with c-JUN**

Functional gene set codes	Functional gene set names	#TFBS sets*
KEGG PATHWAYS IN CANCER (KEGG)	Pathways in cancer	8
GO:0000165 (GOBP)	MAPKKK cascade	6
GO:0043086 (GOBP)	Negative regulation of catalytic activity	5
GO:0031012 (GOCC)	Extracellular matrix	4
GO:0045859 (GOBP)	Regulation of protein kinase activity	4
KEGG MAPK SIGNALING PATHWAY (KEGG)	MAPK signaling pathway	4
GO:0001501 (GOBP)	Skeletal system development	3
GO:0001525 (GOBP)	Angiogenesis	3
GO:0007409 (GOBP)	Axonogenesis	3
GO:0018193 (GOBP)	Peptidyl-amino acid modification	3
GO:0030334 (GOBP)	Regulation of cell migration	3
GO:0032270 (GOBP)	Positive regulation of cellular protein metabolic process	3
GO:0043066 (GOBP)	Negative regulation of apoptosis	3
GO:0048812 (GOBP)	Neuron projection morphogenesis	3

*The number of c-JUN TFBS gene sets associated with each functional gene set.

### Analysis results for the EMT gene set

We now illustrate how to analyze a functional gene set using REGNET. Entering the keyword ‘mesen’ from the ‘Keyword Search’ page, nine similar functional gene sets were presented. Among them, we chose the first one, i.e. the ‘epithelial to mesenchymal transition (GO:0001837)’. This set is associated with eight TFs (MAZ, FOXO4, PITX2, SP1, NFAT, PAX4, ZEB1 and LEF1). Except for MAZ and PAX4, each TF is reported to regulate EMT process. We then investigated the conditions under which each TF might regulate the EMT process. Many putative conditions that are likely to trigger EMT were observed. For example, PCDH24-expressing HCT116 cells shown to abolish tumor formation *in vivo* have ~2000 differentially expressed genes. These expression changes have been confirmed to be quite similar to EMT via further proteomics analysis using 2-DE/MS (GSE10650). Our predictions on EMT suggest MAZ as a candidate TF to regulate EMT in the HCT116 cells. Among the candidate targets of MAZ in the EMT gene set, *Bmp7*, *Col1a1*, *Sfrp2*, *Wnt2* and *Wnt5a* exhibit two or higher expression fold-changes. Interestingly, four of the targets except for Sfrp2 were also captured among the PAX4 results for the same cell condition. This indicates PAX4 and MAZ possibly cooperate on their four common targets to regulate EMT in the HCT cells. For another example, the embroid body cells (GSE9196) the differentiation of which is known to accompany EMT [20] also exhibit clear activation in many of the MAZ targets (Additional file 1: Figure S3a).

**Figure 5:**
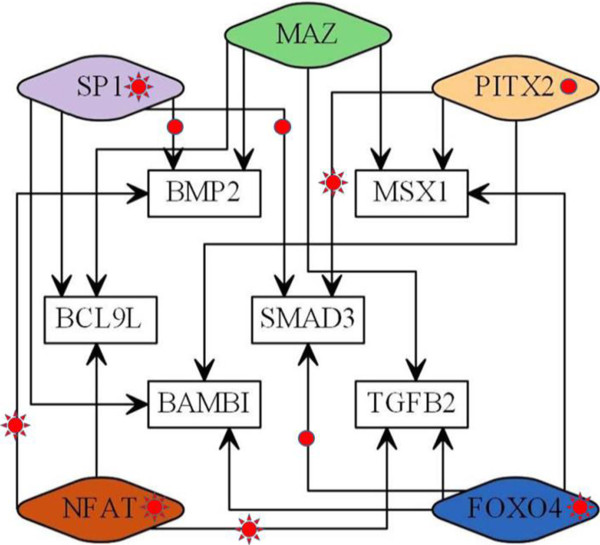
**EMT transcription networks in HUVECs inferred from REGNET.** The sun symbols indicate the corresponding TFs or TF-target relationships have strong supports from the literature. The circle symbols indicate weak supports from the literature. The evidences from the literature are all summarized in Additional file 1: Table S2. The graph is depicted using the Graphviz software.

Among the conditions, HUVECs treated with TNF-alpha are captured by five of the eight TF results (Additional file 1: Figure S3b-e). By mapping each TF to the targets that exhibit two or higher fold-changes, the EMT transcription networks in HUVECs treated with TNF-alpha were constructed that comprised five TFs and six target genes (Figure 5). TNF-alpha is a key cytokine involved in inflammation and cancer, and is known to induce EMT [21]. Because HUVECs do not contain epithelial cells, such EMT signals may indicate ‘endothelial’ to mesenchymal transitions.

Although each regulatory relationship in the networks needs to be validated, it is noteworthy that the predicted edges of the networks were evenly distributed among the five TFs. This implies that the EMT in HUVECs is processed by a tight cooperation of these regulators. Some of the reports for the predicted regulatory relationships are provided in Additional file 1: Table S2.

### A global assessment of REGNET

Because the predicted TNs in REGNET are context-specific (the targets or effects of a TF can change depending on the cell conditions), reliable gold-standard TNs for a global assessment are rarely available. For example, each ChIP-seq or ChIP-chip dataset is also obtained under a specific cell condition and may not serve as a general gold-standard set. Therefore, instead of considering individual cell conditions, we merged all the context-specific targets of a TF for our prediction set (the union of the intersection parts in Figure 2a), and merged multiple ChIP-seq or ChIP-chip data for gold-standard targets of a TF. Among the prediction set, we further selected candidate targets that exhibited ±1.5 or higher fold expression changes across 0.3 ×*k* conditions. We validated the predicted targets of E2F1, JUN and TP53. Because these TFs have multiple known binding sites in MSigDB, we merged all the corresponding targets for each TF. The targets reported from ChIP data are downloaded from ChEA curated database [22]. Three, one and six ChIP datasets were available for E2F1, JUN and TP53, respectively and corresponding targets were merged for each TF. These targets with known TFBS were further selected for true positive gold standard sets.

Among the 564 genes with an E2F1 binding site, 65 were the predicted as context-specific targets. Among the 270 true positive set, 39 were included in the 65 predicted set. This corresponds to specificity 0.9116 and sensitivity 0.1444 with a significant p-value 0.0256 against a random prediction (hypergeometric distribution). The true positive set used here is obtained from a collection of high-throughput ChIP datasets and is missing the three targets Dusp6, Mll5 and Gadd45b, while these targets were identified from our ChIP assay. If we include these three in the positive set, we have an improved sensitivity 0.1538 with a significant p-value 0.0039. Unfortunately, JUN had only one ChIP data in ChEA database. Based on this single ChIP dataset, we had specificity 0.7853, sensitivity 0.2593 but with a less significant p-value 0.1734. Lastly, TP53 had six ChIP datasets. Among the 426 genes with an P53 binding site, 142 were true positives and the 50 predicted targets included 25 true positives. This corresponds to specificity 0.9120, sensitivity 0.1761 and a significant p-value 0.0071.

In summary, REGNET provides highly specific predictions for context-specific transcriptional regulations. Though less sensitive, they still contain a number of novel regulatory relationships as shown in our ChIP assay and the EMT networks.

### Assessment for extended GO and KEGG annotations

Because many genes are still unannotated in GO and KEGG, transcriptional regulations for such genes are not covered by REGNET. To ameliorate the problem, we extended GO and KEGG annotations using protein-protein interaction networks [23], and tested how the extended annotations affect the predictions in REGNET. The annotations are extended as follows: For each gene *g*_*i*_ (protein), we identified the set of interacting proteins *L*_*i*_ from the BioGrid database [24]. For each functional gene set (GO or KEGG annotation), say *F*_*j*_, we assessed the significance of overlap between *L*_*i*_ and F_*j*_ using hypergeometric distribution. If an overlap p-value ≤ p_0_, we assign the annotation *F*_*j*_ to the gene *g*_*i*_.

In the original GO and KEGG annotations, 2,409 GO biological process terms (GO-BP), 369 GO cellular component terms (GO-CC), 186 GO molecular function terms (GO-MF) and 666 KEGG terms are used to annotate genes 164,562, 26,270, 12,875 and 41,063 times respectively. When a threshold p_0_ = 10*E*-_4_ is applied, GO-BP, GO-CC, GO-MF and KEGG terms are used 233,878, 37,455, 23,370 and 54,142 times exhibiting overall 29.8% increase. Using these extended annotations, we regenerated significant patterns in REGNET and extracted context-specific targets of E2F1 and TP53, respectively. With the original annotations, 65 and 50 context-specific targets were identified for E2F1 and TP53, but the extended annotations increased these numbers to 183 and 71, respectively. The extended predictions, as expected, exhibited somewhat reduced specificities 0.7313 and 0.8627, and increased sensitivities 0.3852 and 0.2253 for E2F1 and TP53, respectively.

Here, we illustrated how current annotations can be extended using protein interaction data and how such extension could affect the predictions in REGNET, but such extensions are not included in the current REGNET system.

## Discussion and conclusions

For a given TF or a functional gene set, REGNET provides the associated TNs as well as the corresponding conditions. From the heat map provided for each of the associated pairs of a TF and functional gene set, investigators can select putative targets that exhibit large expression changes across a number of different conditions. We note that the transcription networks inferred in our approach have multiple lines of evidences and a substantial portion of such predictions are highly likely true as validated by reports in the literature and the ChIP assay in the finding reported here.

For a global validation, we pooled targets from multiple ChIP datasets and pooled context-specific predictions for each of three TFs. As expected, predictions in REGNET were highly specific and less sensitive. For example, REGNET provides 65 context-specific targets of E2F1, 42 of which were actually bound by the TF. More importantly, REGNET suggests the cell conditions in which such regulation patterns occur. Despite the high specificity, predictions by REGNET included many novel targets providing useful clues for unraveling cellular processes.

Because many microarray datasets have only a small number of samples, we applied a gene randomizing gene set analysis method [25, 26] to assess the overall expression changes in each gene set. Most gene-randomizing methods, however, suffer from increased false-positives [26]. To reduce false positives, we applied an auxiliary filter that requires a significant regulation pattern to be observed across a number of different conditions.

Specifically, REGNET deploys a composite gene-set analysis method [9, 10] to combine three sources of genomic information: TFBS gene set, functional gene set and gene expression fold-change profile. As a result, three-dimensional information among the TFs, their functional targets and cell conditions are readily explored. Most other approaches including DAVID [27], the Molecular Concept Map [28] and ConceptGen [29] investigate the ‘binary’ relationships between a TFBS gene set and another gene list. One may apply the overlap of gene-sets once more to investigate such three-dimensional relationships, but the repeated use of gene-set overlaps may cause a serious loss of power due to the use of threshold values [26, 30].

Because the activity of a TF is estimated by the overall pattern of expression change in its target genes, REGNET is most useful for finding relatively strong transcriptional activities in which a TF regulates multiple target genes simultaneously. On the other hand, specific regulation of one or two targets that have only small expression changes might be missed in this approach. Another limitation is that by using the TFBS information instead of genome-wide ChIP data, which are mostly unavailable, we only consider the sequence-specific transcriptional regulation. Furthermore, many gene functions are still unknown and remain unannotated in GO or KEGG. We here demonstrated a method to extend annotation gene-sets using protein interaction data and its effect on the performance of REGNET. More reliable extension of gene annotation may be possible by integrating diverse types of genomic data and network information which may help identifying missing transcriptional associations in REGNET [31–33].

Over the last decade, more than a million gene expression microarray datasets have been deposited in public databases [11, 34, 35], covering virtually all of the cell conditions of interest including a broad range of tissues, diseases, development and treatments. Most of them are made up of test and control samples, and their fold-change values provide information on the TNs specific to each condition. For this reason, we have collected the fold-change profiles for a diverse array of cell conditions in order to identify context-specific TNs. We have collected 2,482 human expression fold-change profiles in our current database. As this number increases, it is expected that more specific TNs are newly identified. Moreover, developing a similar system for other species would be valuable for the purpose of comparative analysis.

## Electronic supplementary material

Additional file 1: **The description on ChIP assay and EMT networks.** **Figure S1.** ChIP assay design. **Figure S2.** Heat maps associated with c-JUN. **Figure S3.** EMT transcription networks by five TFs. **Table S1.** Oligos used for ChIP assays. **Table S2.** Evidences of the EMT transcription networks from literature. (PDF 1 MB)

## Abbreviations

TF: Transcription factor
TFBS: Transcription factor binding site
TN: Transcription network
ChIP: Chromatin immunoprecipitation
GO: Gene ontology
KEGG: Kyoto encyclopedia of genes and genomes
MSigDB: Molecular signature database
EMT: Epithelial to mesenchymal transition.
