# Supplementary material for: REGNET: mining context-specific human transcription networks using composite genomic information
Source: BMC Genomics. 2014 Jun 9;15(1):450. doi: 10.1186/1471-2164-15-450 (PMC4070555; doi:10.1186/1471-2164-15-450)
Supplement: Supplementary file 1 — Additional file 1: The description on ChIP assay and EMT networks. Figure S1. ChIP assay design. Figure S2. Heat maps associated with c-JUN. Figure S3. EMT transcription networks by five TFs. Table S1. Oligos used for ChIP assays. Table S2. Evidences of the EMT transcription networks from literature. (PDF 1 MB) [file 12864_2013_6133_MOESM1_ESM.pdf]

Supplementary Material

Quantitative real-time PCR for ChIP

3 ul out of 200ul of purified genomic DNA pulled down with E2F1 antibody was used as a template for quantitative real-time PCR with conditions: 95°C for two minutes followed by 40 cycles at 95°C for 15 s and 60°C for 31 seconds. The PCR was carried in LightCycler 480II (ROCHE) using the premixed 2× real-time SYBR Green reagent (Roche).

Total RNA was isolated from HeLa after the Trizol method (Invitrogen). cDNA was synthesized by cDNA superscript kit (Bio-Rad) and used for quantitative reverse transcription-PCR (RT-PCR) with a LightCycler 480II (ROCHE) real-time PCR detection system. mRNA levels were normalized for expression of *Gapdh* mRNA as a control and calculated by the comparative threshold cycle method.

EMT transcription networks

The EMT transcription networks in Figure 5 are constructed from the five heat maps in Figure S3 (yellow boxes). Four of the five TFs are found to be related to EMT directly or indirectly. Moreover, some of the predicted TF-target relationships are also found in the literature (Table S2)

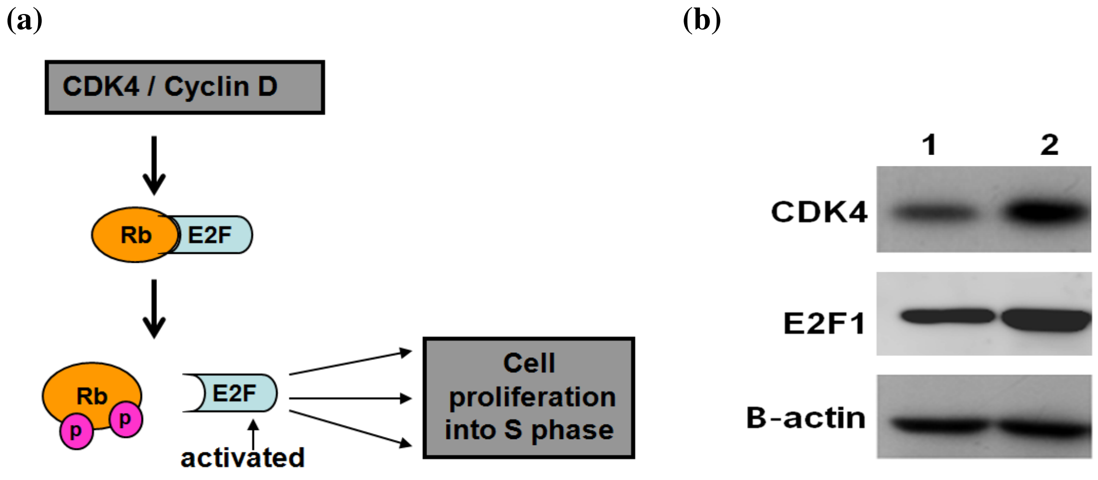

Figure S1. Activating E2F1 in HeLa cells. (a) Action mode of CDK4– E2F1 pathway. The CDK4-Rb-E2F1 pathway regulates the proliferation into S phase. The retinoblastoma (Rb) protein negatively regulates the cell cycle by sequestering E2F family transcription factors. Cyclin-dependent kinase4 (CDK4) promotes S-phase progression and mitosis by sequentially phosphorylating Rb on several serine/threonine residues, thereby rendering the protein inactive [1]. This leads to the release of E2F transcription factors and results in the activation and transcription of E2F responsive genes required for cell proliferation. (b) Western blot for CDK4 and E2F1in HeLa cell line. Low expression of the CDK4 protein was matched with low protein expression of E2F1(B,1) in HeLa cells. After the cells were incubated more than 12 hrs, E2F1 was enriched more than #1(B,2)

Table S1. Oligos used for ChIP assays

Target genes	ChIP primers	mRNA Primers
GADD45B	F:CTTTGCAACCTCCCACTTTC<br>R:CCTCTCTGTTCCCGTCTGAG	F:CTTTGCVAACCTCCCACTTTC<br>R:CCTCTCTGTTCCCGTCTGAG
DUSP6	F:ATCTCTATGGACCCGGGAGT<br>R:CAGGGAGGCATGTCAGAGG	F:CTCCATCCGGCTTCCAAT<br>R:ACTCCCGGGTCCATAGAGAT
BMP2	F:TCCAGCCTGCTGTTTTCTTT<br>R:GGCCCGTTATTCAACTTTCA	F: CTTCTAGCGTTGCTGCTTCC<br>R: AGTGCCTGCGATACAGGTCT
FOSL1	F:GGAAAGACCTCACTCCACGA<br>R:TGCCCATACATGGTGTAACTTC	F: AGTCAGGAGCTGCAGTGGAT<br>R: GGGCTGATCTGTTCACAAGG
MLL5	F:CCCGAGGCCAAGAGAAGTAT<br>R:GAGCTGGCGAGTCTAGTGCT	F: GGGGGTTGATACAGCAGAGA<br>R: GAGGAGGACGAGCACCATAA
NR4A3	F:AGCAGCACTCCTCCAACTCT<br>R:GAGGGAGGGTGTGTGTGTTC	F:CCCCTCCAGGTTCCAGTTAT<br>R:ATTTGGTACACGCAGGAAGG
EPC1	F:TGGGTGCTATTGTCTTGCAG<br>R:GCCAGAAGACTCTGGGGTTA	F: AAGATGCCAAAGCAGGCTCAT<br>R: CAGACTGACTGGCTGCTGAC
MYC	F:CCTCCCATATTCTCCCGTCT<br>R:TGTGTCCTGTTCCAGAG	F:TTCGGGTAGTGGAAAACCAG<br>R:CAGCAGCTCGAATTTCTTCC
E2F3	F:TCAAGGAGGCCTATGCAAAT<br>R:TGCAGGGATACGGTTTACGC	F:CACTTCCACCACCTCCTGTT<br>R:TGACCGCTTTCTCCTAGCTC
GAPDH	F:TCGAAACAGGAGCAGAAGCGA<br>R:TCGAACAGGAGGAGCAGAGAGCGA	F: AATCCACTTACACGCTCATCC<br>R:GACCAGGAGACAATGCAGGT

(a)

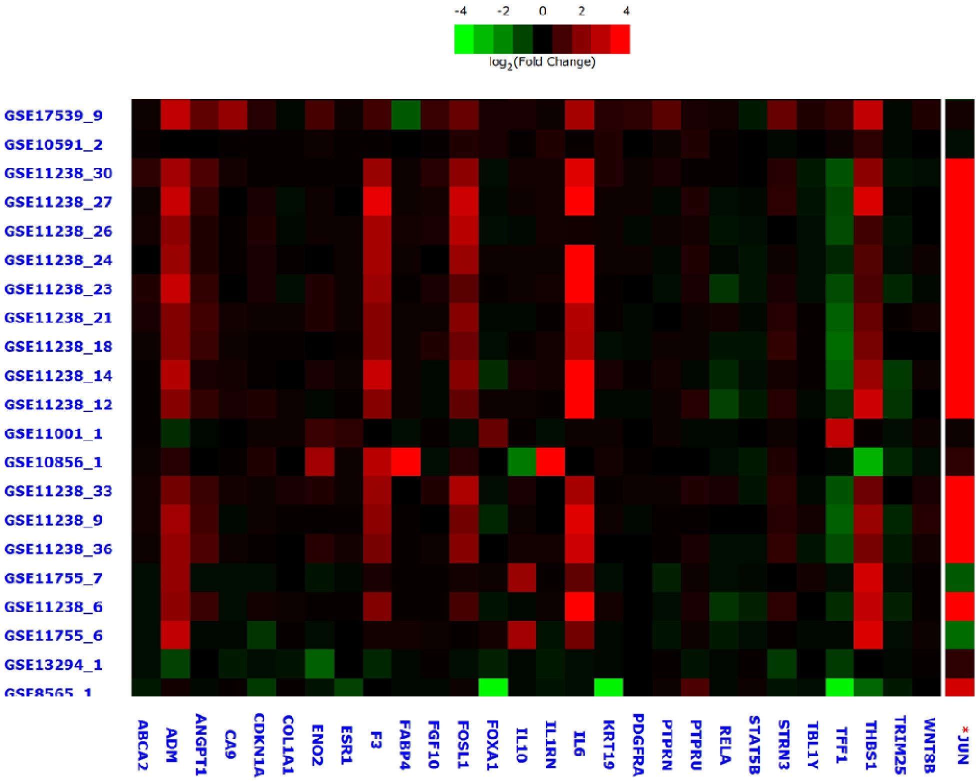
-4 -2 0 2 4
log2(Fold Change)
GSE17539_9
GSE10591_2
GSE11238_30
GSE11238_27
GSE11238_26
GSE11238_24
GSE11238_23
GSE11238_21
GSE11238_18
GSE11238_14
GSE11238_12
GSE11001_1
GSE10856_1
GSE11238_33
GSE11238_9
GSE11238_36
GSE11755_7
GSE11238_6
GSE11755_6
GSE13294_1
ABCA2
ADM
ANGPT1
CA9
CDKN1A
COL1A1
ENO2
ESR1
F3
FABP4
FGF10
FOSL1
FOXA1
IL10
IL1RN
IL6
KRT19
PDGFRA
PTPRN
PTPRU
RELA
STAT5B
STRN3
TBL1Y
TFF1
THBS1
TRIM25
WNT8B
*JUN

(b)

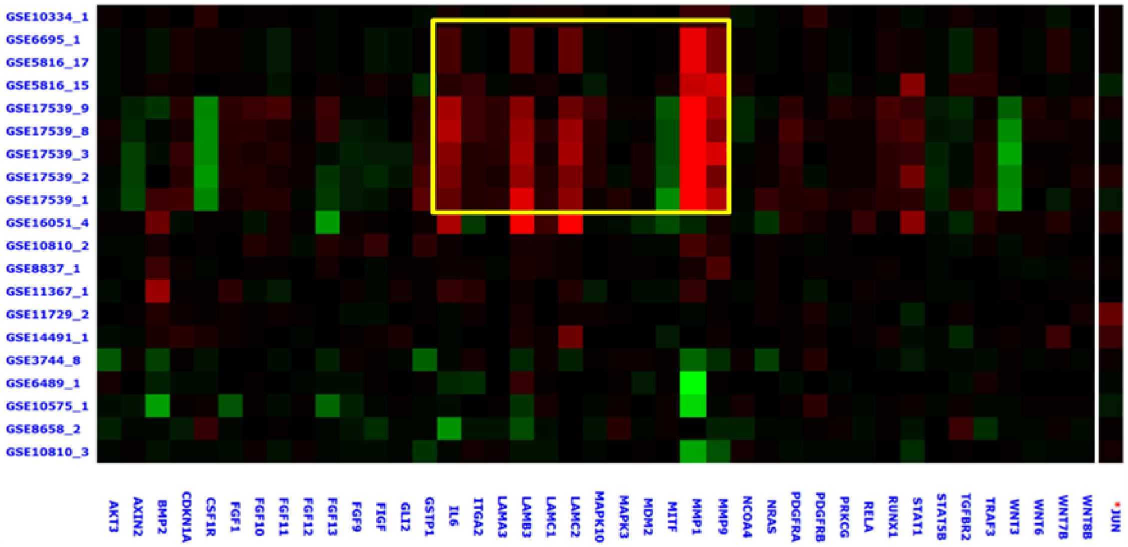
GSE10334_1
GSE6695_1
GSE5816_17
GSE5816_15
GSE17539_9
GSE17539_8
GSE17539_3
GSE17539_2
GSE17539_1
GSE16051_4
GSE10810_2
GSE8837_1
GSE11367_1
GSE11729_2
GSE14491_1
GSE3744_8
GSE6489_1
GSE10575_1
GSE8658_2
GSE10810_3
AKT3
AXIN2
BMP2
CDKN1A
CSF1R
FGF1
FGF10
FGF11
FGF12
FGF13
FGF9
FIGF
GLI2
GSTP1
IL6
ITGA2
LAMA3
LAMB3
LAMC1
LAMC2
MAPK10
MAPK3
MDM2
MITF
MMP1
MMP9
NCOA4
NRAS
PDGFRA
PDGFRB
PRKCG
RELA
RUNX1
STAT1
STAT5B
TGFBR2
TRAF3
WNT3
WNT6
WNT7B
WNT8B
*JUN

(c)

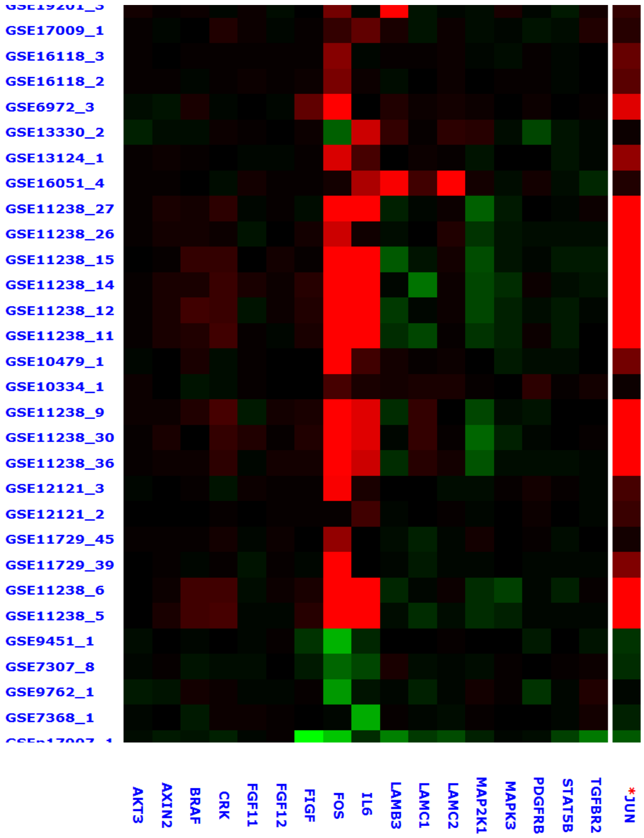

Figure S2. (a) The heat map for the association between c-JUN and the gene set 'steroid hormone stimulus (GO:0048545)'. A part of the 56 conditions that associate c-JUN and the GO gene set are shown. Five target genes, *Adm*, *F3*, *Fosl1*, *Il6* and *Thbs1* exhibit clear activation across a number of virus-treated HeLa cells. (b) Transcription networks for an c-JUN TFBS gene set, TGANTCA_V$AP1_C and 'pathways in cancer (KEGG)' gene set. The red stripes in the yellow box indicate the five well-known c-JUN targets, *Il6*, *Lamb3*, *Lamc2*, *Mmp1* and *Mmp9* are highly activated under 5-aza-2-deoxycytidine treatment on an non-small cell lung cancer cell line and several of the virus-treated HeLa cell conditions. (c) Transcription networks for another c-JUN TFBS gene set, V$AP1FJ_Q2 and 'pathways in cancer (KEGG)' gene set. *Fos* and *Il6* exhibit strong co-expression pattern with Jun.

Table S2. Evidences of the EMT transcription networks in Figure 5 from literature.

Transcription factors (relation to EMT)	Predicted targets (relationship to TF)
SP1 [2]	*Bmp2* [3]
NFAT [4]	*Bmp* [5], *Tgfb* [6]
FOXO4 [7]	*Smad3* (complex, MIPS)
PITX2 [8]	*Smad3* [9]
	Msx1 [8]
MAZ	

(a)

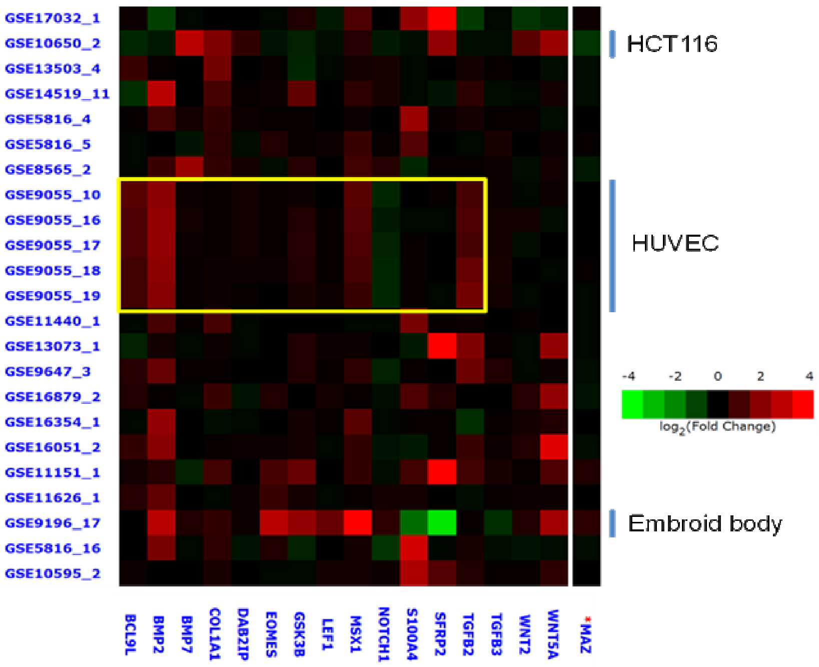

(b)

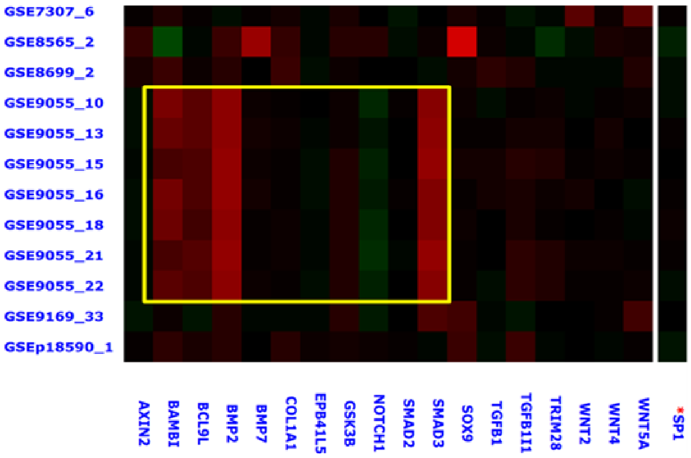

(c)

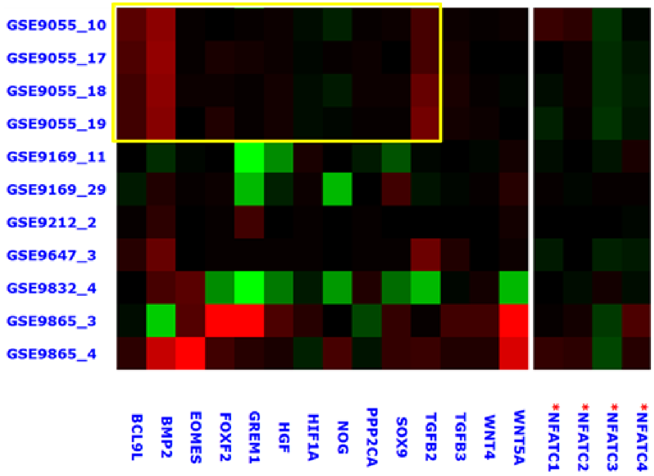

(d)

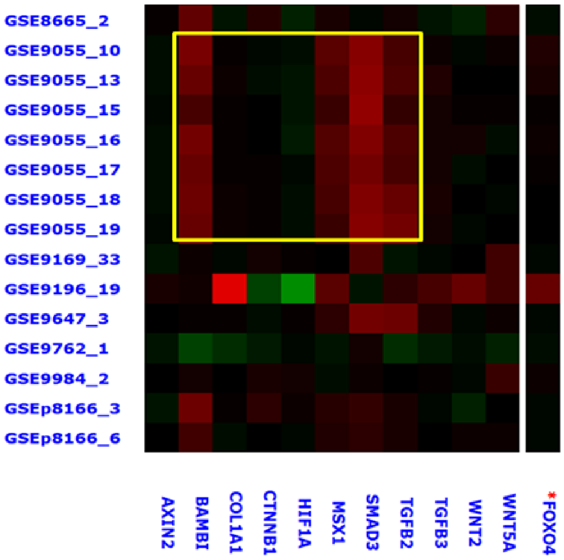

(e)

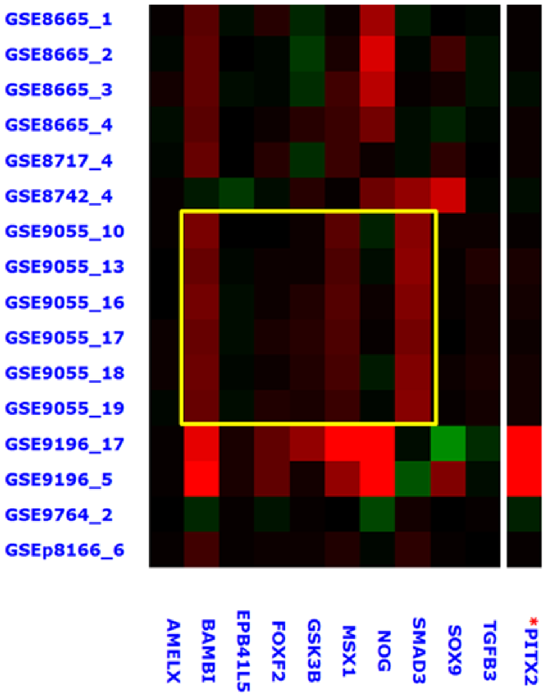

Figure S3 **(a)** EMT transcription network by MAZ. Several MAZ targets are highly activated in HCT 116 cells and Embroid body cells. Four targets *Bcl9l*, *Msx1*, and *Tgfb2* are relatively highly activated in HUVECs treated with TNF-alpha. **(b)** HUVEC EMT network for SP1. Four targets *Bambi*, *Bcl9l*, *BMP2*, and *Smad3* are highly activated. **(c)** HUVEC EMT network for NFAT. Three targets *Bcl9l*, *Bmp2*, and *Tgfb2* are relatively highly activated. **(d)** HUVEC EMT network for FOXO4. Four targets *Bambi*, *Msx1*, *Smad3*, and *Tgfb2* are highly activated. **(e)** HUVEC EMT network for PITX2. Three targets *Bambi*, *Msx1*, and *Smad3* are relatively highly activated.

Supplementary References

1. Satyanarayana A, Kaldis P: **Mammalian cell-cycle regulation: several Cdks, numerous cyclins and diverse compensatory mechanisms**. *Oncogene* 2009, **28**(33):2925-2939.
2. Jungert K, Buck A, von Wichert G, Adler G, Konig A, Buchholz M, Gress TM, Ellenrieder V: **Sp1 is required for transforming growth factor-beta-induced mesenchymal transition and migration in pancreatic cancer cells**. *Cancer Res* 2007, **67**(4):1563-1570.
3. Xu J, Rogers MB: **Modulation of Bone Morphogenetic Protein (BMP) 2 gene expression by Sp1 transcription factors**. *Gene* 2007, **392**(1-2):221-229.
4. Mancini M, Toker A: **NFAT proteins: emerging roles in cancer progression**. *Nat Rev Cancer* 2009, **9**(11):810-820.
5. Tomita M, Reinhold MI, Molkentin JD, Naski MC: **Calcineurin and NFAT4 induce chondrogenesis**. *J Biol Chem* 2002, **277**(44):42214-42218.
6. Singh G, Singh SK, Konig A, Reutlinger K, Nye MD, Adhikary T, Eilers M, Gress TM, Fernandez-Zapico ME, Ellenrieder V: **Sequential activation of NFAT and c-Myc transcription factors mediates the TGF-beta switch from a suppressor to a promoter of cancer cell proliferation**. *J Biol Chem* 2010, **285**(35):27241-27250.
7. Lee MJ, Yu GR, Yoo HJ, Kim JH, Yoon BI, Choi YK, Kim DG: **ANXA8 down-regulation by EGF-FOXO4 signaling is involved in cell scattering and tumor metastasis of cholangiocarcinoma**. *Gastroenterology* 2009, **137**(3):1138-1150, 1150 e1131-1139.
8. Chen YH, Ishii M, Sucov HM, Maxson RE, Jr.: **Msx1 and Msx2 are required for endothelial-mesenchymal transformation of the atrioventricular cushions and patterning of the atrioventricular myocardium**. *BMC Dev Biol* 2008, **8**:75.
9. Suszko MI, Antenos M, Balkin DM, Woodruff TK: **Smad3 and Pitx2 cooperate in stimulation of FSHbeta gene transcription**. *Mol Cell Endocrinol* 2008, **281**(1-2):27-36.
